# An Updated Overview on the Role of Small Molecules and Natural Compounds in the “Young Science” of Rejuvenation

**DOI:** 10.3390/antiox12020288

**Published:** 2023-01-27

**Authors:** Giovanni Ribaudo, Alessandra Gianoncelli

**Affiliations:** Department of Molecular and Translational Medicine, University of Brescia, 25121 Brescia, Italy

**Keywords:** rejuvenation, aging clocks, G-quadruplex, senotherapeutic, antioxidants, natural compounds

## Abstract

Aging is a gradual process that occurs over time which leads to a progressive decline of cells and tissues. Telomere shortening, genetic instability, epigenetic alteration, and the accumulation of misfolded proteins represent the main hallmarks that cause perturbed cellular functions; this occurs in conjunction with the progression of the so-called “aging clocks”. Rejuvenation aims to influence the natural evolution of such aging clocks and to enhance regenerative capacity, thus overcoming the limitations of common anti-aging interventions. Current rejuvenation processes are based on heterochronic parabiosis, cell damage dilution through asymmetrical cell division, the excretion of extracellular vesicles, the modulation of genetic instability involving G-quadruplexes and DNA methylation, and cell reprogramming using Yamanaka factors and the actions of antioxidant species. In this context, we reviewed the most recent contributions that report on small molecules acting as senotherapeutics; these molecules act by promoting one or more of the abovementioned processes. Candidate drugs and natural compounds that are being studied as potential rejuvenation therapies act by interfering with CDGSH iron-sulfur domain 2 (CISD2) expression, G-quadruplex structures, DNA methylation, and mitochondrial decay. Moreover, direct and indirect antioxidants have been reported to counteract or revert aging through a combination of mixed mechanisms.

## 1. Introduction

Aging is a process of gradual decline which occurs over time. It can be observed in cells and tissues in almost all organisms. It progressively leads to a loss of physiological integrity, impaired function, and an increased vulnerability to death [1,2].

As highlighted by López-Otín et al., aging is characterized by molecular hallmarks such as: genomic instability, telomere attrition, epigenetic alterations, loss of proteostasis, deregulated nutrient-sensing, mitochondrial dysfunction, cellular senescence, stem cell exhaustion, and altered intercellular communication [2].

Perturbed cellular functions occur in conjunction with the progression of aging-related changes that can be measured by so-called “aging clocks” [1].

Cell and organism lifespans are pre-defined in accordance with the theory of “programmed aging”, which is based on different hypotheses [3]. First, concerning genetically programmed longevity, aging could be the consequence of the expression, or the lack of expression, of certain genes; this involves events related to genetic instability, such as the shortening of telomeres. The second hypothesis argues that aging could be regulated by endocrine mechanisms, through which, hormone release would be able to act on biological clocks [4]. Finally, the third hypothesis is based on the concept of immunosenescence, which argues that the immune system decreases in terms of efficacy during the aging process, thus increasing an organism’s susceptibility to diseases [3]. Nowadays, transcriptomic and epigenetic modifications are believed to be reversible; this has invigorated the search for strategies that rejuvenate cells by causing molecular changes [1].

**Figure 1:**
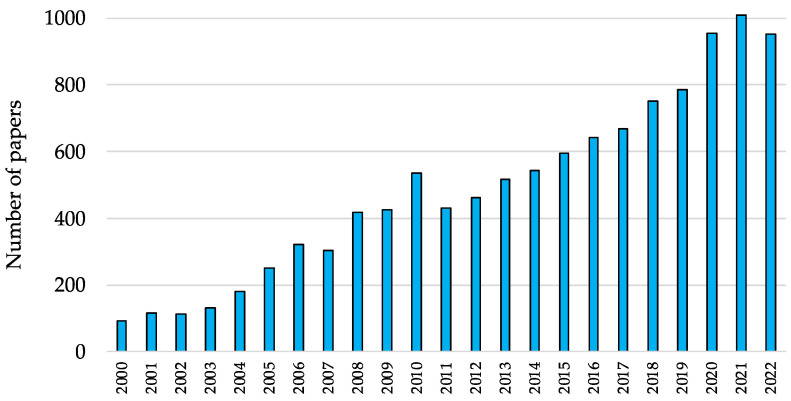
Number of papers published between 2000 and 2022, which were retrieved using a literature search on PubMed (www.ncbi.nlm.nih.gov/pubmed/) with the query term “rejuvenation”.

Defining rejuvenation is a rather debated subject, as will be discussed in Section 2 of this review; however, emerging contributions to the field suggest that biological aging can be reversed rather than just attenuated [5]. Although current rejuvenation studies mainly rely on preliminary, albeit encouraging, in vitro evidence, several venture capitalists, cryptocurrency companies, and tech investors are investing in biotech startups that are conducting basic research on epigenetic reprogramming [6]. As a result, the interest in this form of “young science” is propelling the academic research on this subject further, as is evident by the increasing number of scientific reports (Figure 1).

More than 80 research papers and reviews were considered in the current article. Scientific contributions were retrieved by searching PubMed (www.ncbi.nlm.nih.gov/pubmed/) and Scopus (www.scopus.com) databases; keywords such as “rejuvenation”, “rejuvenating”, “anti-aging”, “senescence”, “small molecules”, “natural compounds”, and combinations of these terms were used to retrieve relevant articles. To provide readers with the most updated overview possible, only papers published in 2022 were considered in the first round of searching the literature. The database search was then expanded to include relevant secondary references that were cited in the retrieved reports. The 3D models of the studied macromolecular targets and complexes were retrieved from the Protein Data Bank (PDB, www.rcsb.org), and UCSF Chimera software was used to prepare the artworks [7].

## 2. Aging and Rejuvenation

In accordance with the modern definition, “rejuvenation” may be described as the branch of science that aims to influence the natural progression of aging biological clocks with cellular reprogramming, a phenomenon that can be achieved on several levels and with different approaches [8]. Rejuvenation is a concept that is strictly related to anti-aging; however, according to recent reports, definitions relating to rejuvenation cannot be fully implemented.

Aging comprises a series of events affecting cellular processes; this leads to functional decline, increased frailty, and increased susceptibility to chronic disease [9]; thus, aging is commonly referred to as the most significant risk factor for human mortality [5].

During anti-aging interventions, the aim is to maintain or preserve the aging biomarker status; this is achieved by avoiding or slowing down its decline. Conversely, rejuvenation is strongly connected to the enhancement of an organism’s regenerative capacity; ideally, this leads to a reduction in biological age [5].

The main strategies that aim to extend lifespans can be divided into categories. These categories comprise interventions that directly treat the causes of mortality, approaches that slow down or attenuate the biological aging process, and methods that lead to rejuvenation; in other words, the reversal of aging processes. The latter category has been deemed unrealistic in the past; however, recent reports have demonstrated, through proven readout data, chiefly in the form of different biomarkers, that putative rejuvenation therapies can achieve age reversal [5]. Thus, it must be clarified that rejuvenation represents only one of the mechanisms through which lifespan extension can be achieved; however, these two expressions cannot be considered as having the same meaning.

The first aspect that must be discussed concerns the difference between longevity interventions, which aim to slow down aging, and rejuvenation therapies. As reported by Zhang et al., a major change in perspective is needed when approaching the concept of rejuvenation. In fact, the aging of mammalians has been traditionally defined as an irreversible process [5]. This is mainly due to the fact that most cellular structures are irreplaceable, and although certain body parts or organs can be regenerated in some animal species, human adults lack proper cross-tissue regeneration processes [10]. On the other hand, it is also true that the abovementioned paradigm has been challenged by recent findings. Indeed, reports in the literature have shown that axon regeneration, the recovery of eyesight, and thymus regeneration can be achieved in animal models through cellular reprogramming [5,11], a strategy which will be discussed in more detail in the following parts of this review.

To define a process as a “rejuvenation” process, and to further distinguish it from anti-aging interventions, there is a need for clear methods that assess biological age and its dynamics; this is because rejuvenation processes interfere with age-related phenotypes on different levels. From a biological point of view, the considered biomarkers should be noninvasive or nonlethal; importantly, this aspect limits studies at the tissue level [5]. Multi- and single-tissue clocks have been developed and applied to humans in order to measure age and predict health span, lifespan, and mortality risk [5]. Many detectable biological features correlate with chronological age, such as telomere length or the racemization of amino acids [12,13], and the most studied biological feature is likely to be the one that is connected to the measurement of DNA methylation levels [14]. In this context, Hannum et al. published the first example of an epigenetic clock to be represented by 71 CpG sites [15], whereas Horvath reported a more precise and comprehensive multi-tissue aging clock that consisted of 353 CpG sites [14,16].

Data analysis allowed the setup of several “biological aging clocks”; these clocks were created in accordance with information concerning epigenomes, transcriptomes, and immunomes [10,17]. Zhavoronkov et al. recently reviewed state of the art deep aging clocks, where machine learning techniques have been applied to many measurable features, such as changes in time; these features are considered to be biomarkers of aging and aging clocks [18].

In general, even if rejuvenation strategies share commonalities with existing longevity interventions, the latter cannot systemically reverse biological age; rather, they attenuate age-related hallmarks, whereas the former aims to enhance the regenerative capacity of an organism [5].

To put this topic in the proper perspective, we must also consider that rejuvenation is a phenomenon that has already been studied in other organisms, and it is not a phenomenon that is exclusive to mammalians [19]. Some plants grow indefinitely and propagate routinely; this is due to the presence of meristems, which are stem cells that follow the principle of damage dilution [20], a concept that will be exposed in the following paragraphs. This strategy has also been adopted by some bacteria which divide asymmetrically. As a result, some bacteria inherit an old cell wall and others inherit a new one, and it has been observed that the former type of bacteria grow more slowly [21]. In *Saccharomyces cerevisiae*, the rate of division decreases with each subsequent generation, and mother cells that retain denaturated protein aggregates and aging mitochondria have been observed; thus, “younger”, or “rejuvenated”, daughter cells were able to be produced [22].

## 3. Cellular Processes Promoting Rejuvenation

According to Zhang et al., rejuvenation interventions can be classified into three main categories. These categories are: heterochronic transplantation, cellular reprogramming, and early embryonic dynamics. Moreover, the authors identified three major classes of approaches that have been pursued in recently reported rejuvenation strategies: drug interventions, based on small molecules or biotechnologies, to rewind biological age; reprogramming factors that interfere with epigenetic processes; and transient or partial reprogramming, which is arguably the most appealing strategy [5].

Although rejuvenation is considered to be a “young science”, the earliest reports that detail age reversal date back several decades; indeed, it was reported that tissues and organs from animals of one age can be transplanted to animals of different ages to form “heterochronic” chimeras. Consequently, heterochronic parabiosis, obtained through surgical procedures, became a strategy to extend an animal’s lifespan [23,24]. Furthermore, Conboy et al. noted that when the circulatory system of an aged mouse was surgically spliced together with that of a younger mouse, an insurgence of youthful features in the old mouse was observed in the brain, muscle, and liver; this resulted in increased cognitive function, replenished stem cell pools, and augmented regenerative potential [25].

However, one may still argue that the abovementioned observations may still fall within the field of longevity intervention rather than that of true rejuvenation therapies; this is because they are based on a dilution of “old factors”, and damage has still occurred, likely through damaging molecules [5]. Nevertheless, damage dilution is a possible mechanism that is shared by different rejuvenation interventions, and it has been intensively studied. This theory is based on the fact that damaged cell constituents (DCC) accumulate in the aging cell. Such species emerge from different processes, such as various kinds of stress and reactive oxygen species (ROS) which promote damage to lipids, proteins, and nucleic acids. More specifically, oxidative stress, which is the consequence of an imbalance between pro- and antioxidant species, is currently defined as one of the main factors that cause age-related damages [3]. Although oxidative stress damage, as well as other forms of stress damage are often reversible, repair systems are not infallible. Moreover, as a result of the aging process, DCC accumulate in the cell [19]. The damage dilution theory is based on the hypothesis that some cells can escape senescence as they do not divide symmetrically. Asymmetrical cell division (ACD) leads to the partitioning of DCC in a way that ensures one daughter cell inherits most of them, whereas the other daughter cell has significantly fewer DCC; consequently, the latter daughter cell can be described as “rejuvenated” [26]. Stem cells divide by ACD, although they are not completely immune from aging as senescence can occur as a result of mutations, epigenetic changes, and environmental damage [19].

It is also true that there are some cells which divide symmetrically; thus, they are not rejuvenated via ACS, but they can elude senescence. Cancer cells, for example, eliminate DCC via another strategy, which involves the excretion of vesicles [19]. Membrane-protected extracellular vesicles (EVs) can play a role in signaling; however, they can also contain DCC, damaged DNA, and damaged or misfolded proteins. It must be noted that a similar process occurs in bacteria, wherein “minicells” containing damaged and misfolded proteins are excreted [27,28]. Nevertheless, other recent reports suggest that some EVs may act as “traveling metabolic units”, and thus, they may play a positive role in mediating rejuvenation [9].

Similarly, Zhang et al. reported that in mouse models in which hypothalamic stem/progenitor cells were ablated, accelerated aging and shortened lifespans can be observed. On the other hand, anti-aging effects, and most importantly, lifespan extension, were achieved in mice that were implanted with healthy hypothalamic stem/progenitor cells. The authors highlighted the role of the mice’s exosomes, and of exosomal microRNAs (miRNAs), in particular. In fact, treatment with exosomes led to a reduction in the development of aging-associated disorders [29].

Mammalian somatic cells in tissue cultures divide symmetrically, and they die after a limited number of divisions (50–60); this is usually referred to as the “Hayflick limit”. This is connected to the progressive shortening of telomeres and DNA sequences at the ends of chromosomes, which occurs as a result of successive divisions. This event represents a kind of pre-programmed count-down process; indeed, cell division is arrested after the “Hayflick limit” is reached. It has been shown that telomere-dependent aging can be reversed through the activation of the telomerase enzyme, which elongates telomeres; however, this mechanism is also involved in the onset of cancer and its progression as it is connected to cellular immortalization [19].

DNA methylation, as anticipated, can be considered an epigenetic clock. In fact, as a result of aging, DNA, and its associated histone proteins, are progressively methylated to make the cells less prone to division. Demethylation, on the other hand, is seen as a rejuvenation approach [19].

Sirtuins are a class of enzymes that exhibit deacetylase activity; this depends on nicotinamide adenine dinucleotide (NAD^+^) to function. Consequently, NAD^+^ contributes to the regulation of metabolism, the oxidative state, and cell survival; thus, it influences aging process. Additionally, it has been noted that NAD^+^ levels decrease with age, as has also been observed for the other endogenous antioxidant agent, glutathione (GSH) [9].

**Figure 2:**
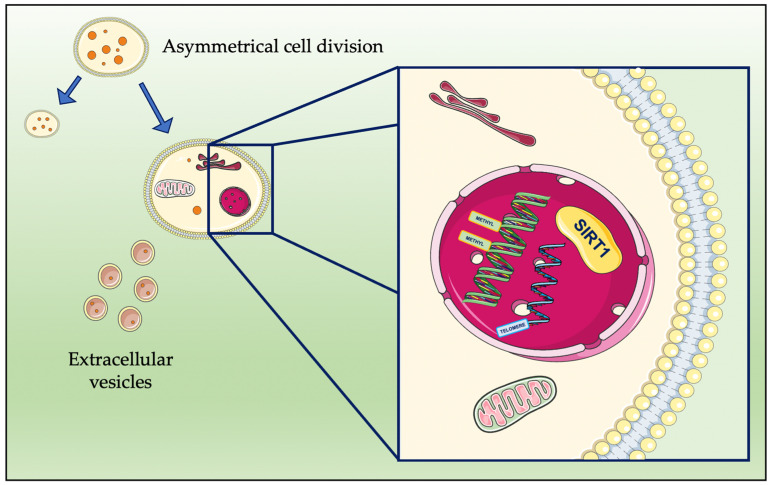
Schematic representation of the cellular events mediating rejuvenation. Asymmetrical cell division and the elimination of extracellular vesicles occur in conjunction with processes that take place in the nucleus; these processes are related to SIRT1 activation, DNA methylation, and telomeres. The artwork was prepared using resources from Servier Medical Art (smart.servier.com, Accessed on 15 January 2023).

A schematic representation of the main processes involved in the abovementioned cellular events that are related to rejuvenation are shown in Figure 2.

Additionally, in the early 2000s, four critical “reprogramming factors” were discovered, named Oct4, Sox2, Klf4, and c-Myc. These “Yamanaka factors”, when expressed in somatic cells, could reverse the developmental status of cells to that of embryos, thus leading to the generation of induced pluripotent stem cells (iPS) [30] and to a reduction in epigenetic age, according to biological clocks. Furthermore, the temporary induction of iPS, followed by the induction of a senescence-associated secretory phenotype (SASP), causes in vivo reprogramming and cellular plasticity in aging animal models. Interleukin-6 appears to be involved in the observed properties relating to plasticity [9,31]. It has been demonstrated that reprogramming can be performed in vitro, as has been shown in human fibroblasts and chondrocytes. Moreover, this has also been demonstrated in vivo, in experiments that aim to extend the lifespans of mice or that aim to reverse age-associated changes in retinal ganglion cells; the latter experiment led to the restoration of vision in a glaucoma mouse model [11,32]. The major drawback of this approach lies in the fact that iPS reprogramming converts somatic cells so that they revert to an embryonic stem cell-like state. This can cause the loss of original cell identity and function; thus, one of the most recent trends involves so-called “transient reprogramming”. During transient programming, Yamanaka factors are expressed for short period of time, thus achieving rejuvenation without loss of cell identity. Moreover, Gill et al. described a transient reprogramming strategy wherein Yamanaka factors are expressed until the maturation phase of reprogramming; then, the induction phase is abolished, thus achieving the conservation of cell identity [1].

## 4. Druggable Rejuvenation Mechanisms

Investigations into the druggable mechanisms that are involved in rejuvenation processes are recent; these reports were largely published in the last decade.

In a clinical trial, Fahy et al. demonstrated that recombinant human growth hormone (rhGH) administration, in the context of a protocol used to regenerate the thymus, can induce protective immunological changes. It can also induce a mean epigenetic age that is approximately 1.5 years less than the baseline after 1 year of treatment. Since it would represents the first experiment which produces results that show an increase in predicted human lifespan, this finding is particularly significant [33].

However, it must also be noted that controversial opinions on the role of GH in the aging process are present in the literature; previous studies concerning animal models showed that the downregulation of the GH/insulin-like growth factor-1 (IGF-1)/insulin pathway could promote lifespan extension; although, the results are contradictory in humans [34]. On the other hand, Mariño et al. showed that treatment with recombinant IGF-1 extends the lifespan of progeroid mice [35]. The literature is also divided on whether GH or IGF-1 exhibits positive or negative effects when placed under oxidative stress conditions; animals with lifelong decreased GH/IGF-1 signaling are protected from oxidative stress, whereas models examining age-induced reductions in GH/IGF-1 signals show increased oxidative stress levels [36].

According to the abovementioned reports, the balance between IGF, GH, and their fine regulation appears to be relevant in terms of longevity, rather than a transient boost in the concentration of one or more mediators. Moreover, both hormones play a dual role as they can promote either cell proliferation or cellular senescence; thus, the dose of GH/IGF-1, duration of GH/IGF-1 exposure, cell type, and tissue type may influence, either alone or in combination, the effect that hormones have on senescence and lifespan [34,36,37].

The field of senotherapeutics, which concerns a class of small molecules with different chemical origins, is more strictly connected to pharmacological interventions against age-related functional decline in nervous and cardiovascular systems [5,38]. More precisely, such compounds are currently classified into senolytics, which eliminate senescent cells, and senomorphics, that damper their secretome [9].

Senotherapeutic drugs delay the progression of senescence and tissue dysfunction. Ideally, they would be used in therapeutic strategies that work against age-related disorders; more specifically, they would be used to rejuvenate cells in the fields of regenerative medicine, tissue repair, and transplantation [38]. Wong et al. recently reviewed the compounds that have been studied as senotherapeutics, a group of more than 20 compounds that includes natural molecules such as quercetin, resveratrol, artemisinin, and melatonin, as well as marketed drugs such as dasatinib or metformin [38].

In the next part of this review, an overview of the main rejuvenation mechanisms, which are targeted by small molecules of natural or synthetic origin, is presented.

### 4.1. CDGSH Iron-Sulfur Domain 2 (CISD2)

**Figure 3:**
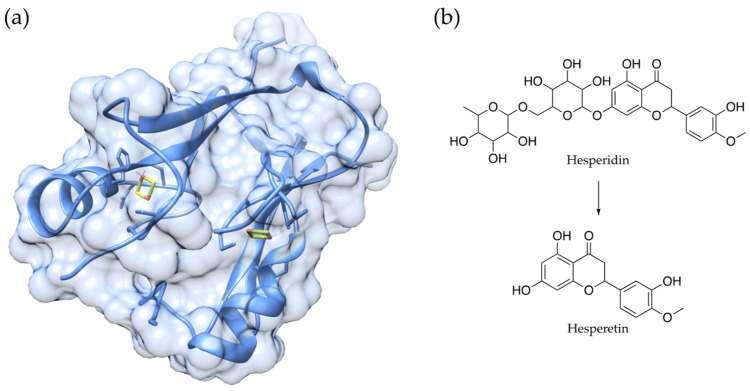
Three-dimensional structure of Miner1 (**a**), encoded by the CISD2 gene (PDB ID 3FNV), and chemical structures of the natural compound hesperidin, and its derivative hesperetin (**b**), the latter of which has been shown to interfere with gene expression and promote rejuvenation.

Eight genes (BUB1B, CISD2, KLOTHO, PAWR, PPARG, PTEN, SIRT1, and SIRT6) are currently defined as “pro-longevity genes”, in accordance with the Human Aging Genomic Resources; this is because they have experimentally demonstrated that they mediate lifespans in mammals. In particular, it has been shown that higher levels of CDGSH iron-sulfur domain 2 (CISD2) expression slow down aging and promote longevity in mouse models [39]. Physiologically, CISD2 is involved in maintaining mitochondrial function, endoplasmic reticulum integrity, intracellular Ca^2+^ homeostasis, and redox status. The members of the CISD protein family are composed of CDGSH iron-sulfur domains (Figure 3a), and they can be localized in the mitochondrial outer membrane, in the endoplasmic reticulum, and in mitochondrial-associated ER membranes [40,41].

As mentioned above, CISD2 is one of the few pro-longevity genes that have been identified in mammals, and although its expression normally decreases with age, Yeh et al. recently showed that it can be reactivated by using small molecules in mice who are in the later stages of life [39]. Several natural compounds have been proposed and studied as CISD2 activators, including hesperidin, curcumin, α-eleostearic acid, sophoricoside, genistein, and formononetin [42,43,44,45]. Yeh et al. studied the potential of hesperetin (Figure 3b), which is the product of the in vivo biotransformation of hesperidin [39]. Hesperidin and hesperetin are well-known flavonoids that can be found in several *Citrus* fruits; these flavonoids have been studied for their biological activities as antibacterial, anticancer, and anti-inflammatory agents. Most importantly, the compounds have been widely investigated for their antioxidant properties [46].

Hesperetin was highlighted as the most promising CISD2 activator, enhancing gene expression both in vitro and in vivo [45]. Treatment with hesperetin extended the lifespan and improved the long-term health of the mice, thus attenuating whole-body metabolic decline, reducing fat, improving glucose homeostasis, and slowing down muscular aging, as confirmed by RNA sequencing. Importantly, hesperetin lost its beneficial anti-aging effect in CISD2 knock-out mice, and transcriptomic analysis confirmed that most of the differentially expressed genes upon hesperetin administration are CISD2-dependent [39,45].

### 4.2. G-Quadruplexes and DNA Methylation

G-quadruplexes (G4s) are non-canonical secondary structures that are formed by guanine-rich sequences in the genome. G4s can be recognized by specific proteins, including methylation-regulating enzymes [47]. As anticipated, the DNA methylation level is one of the main biological clocks that indicate the extent of aging [19], and recent reports have noted the connection between G4s and aging; this is because sites that are considered to be aging clocks are enriched with G4-forming sequences [48].

In fact, it has been hypothesized that G4s may contribute to disease progression by influencing epigenetic control in the cell. G4-forming sequences are not randomly distributed throughout the genome, as they are mainly located in telomeres and oncogenes [49]. They represent attractive targets for developing novel antiproliferative and anti-infective agents of natural and synthetic origin [50,51,52]. In general, the stabilization of the G4 structures using small molecules can regulate transcription and translation, either via upregulation or downregulation, depending on the target gene [53]. In the context of neurodegenerative disorders, the role of G4s is highly debated, as they have been proposed as targets that can ameliorate these conditions [54,55].

**Figure 4:**
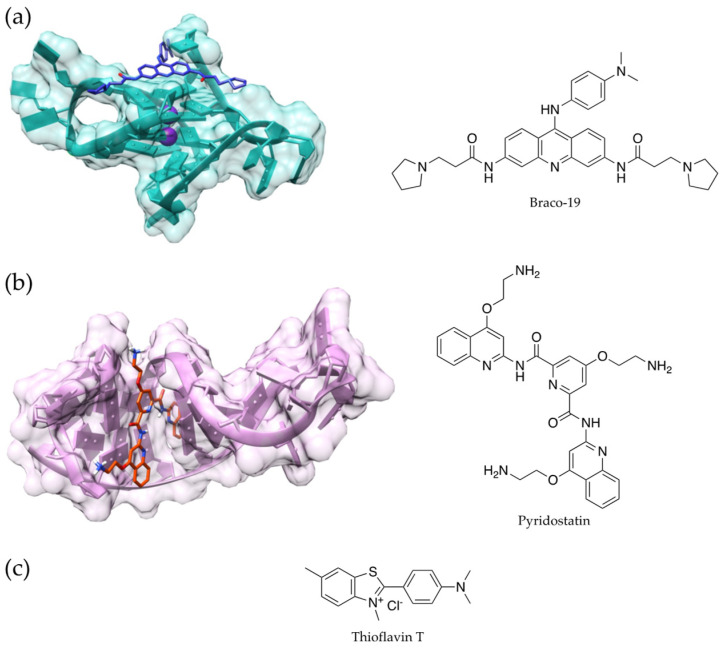
Chemical structure and 3D model of a ligand-G4 DNA complex for Braco-19 (PDB ID 3CE5, (**a**) and pyridostatin (PDB ID 7X3A) (**b**); chemical structure of thioflavin T (**c**).

Nevertheless, as anticipated, Raucchaus et al. reported that G4s are also highly present in terms of human aging clock sites. Most importantly, the authors highlighted the comparable enrichment of G4s in the binding sites of enzymes that operate both methylation and demethylation; this supports the hypothesis that G4s may be more involved in the perturbation of DNA-methylation than in the process that promotes either hyper- or hypomethylation [48]. Similarly, Moruno-Manchon et al. showed that older mice treated with G4 binders (Figure 4a,b) had enhanced senescence-associated phenotypes in their brains, and they exhibited increased cognitive deficits. Additionally, DNA damage was observed in cultured neurons, astrocytes, and microglia [56]. More recently, in a work from the same research group, Noh et al. reported the pro-aging effects of RNA G4s using thioflavin T (Figure 4c) in order to detect the secondary arrangement; in this experimental model, they demonstrated that G4 stabilizers can induce senescence [53].

### 4.3. Mitochondrial Decay and Antioxidants

During the cell aging process, mitochondria undergo a process which is known as “mitochondrial decay”; this is key to the aging process, and for the onset and progression of age-related diseases [22]. Moreover, it must be noted that mitochondria are considered to be the main site where chemical reactions generating ROS occur within the cell [3]. Concerning their underlying mechanisms, mitochondria may be the targets of their own oxidative damage, and the age-related loss of the mitochondrial respiratory capacity is a well-established concept that is based on the reduction of mitochondrial GSH (mtGSH). The loss of mtGSH halts peroxidase activity and stimulates the activation of cell death processes. Mitochondrial decay is connected to oxidative stress and increased DNA methylation levels; thus, it is related to cellular aging [57]. Consequently, the use of antioxidants in vivo has been adopted as a strategy to combat this process; however, limited effects were observed in terms of rejuvenation.

Visioli et al. recently reviewed natural and dietary compounds that target mitochondria, induce gene expression, and enhance mitochondrial biogenesis, mitophagy, or restoring metabolites that naturally decline with age [58].

The limited efficacy of antioxidant supplementation strategies has traditionally been associated with limited bioavailability and a lack of selectivity in terms of previously studied compounds in mitochondria; thus, more recently, bioavailable metabolites have been considered as intervention strategies that can revert mitochondrial decay [58]. For example, given the primary relevance of mtGSH in preventing oxidative stress in mitochondria, N-acetyl-cysteine, which acts as a prodrug to increase GSH synthesis, has been proposed as a potential therapeutic tool [59].

As anticipated, small molecules that directly act as antioxidants exhibit a lack of selectivity and they showed limited efficacy; thus, another strategy involves administering compounds that increase the expression of endogenous antioxidants. The principal mechanism targeted by these molecules involves the activation of the transcriptional regulator, NF-E2-related factor 2 (Nrf2) [60]. Included in the studied compounds in this class are sulforaphane, lipoic acid, caffeic acid, quercetin, fisetin, and naringenin [58].

**Figure 5:**
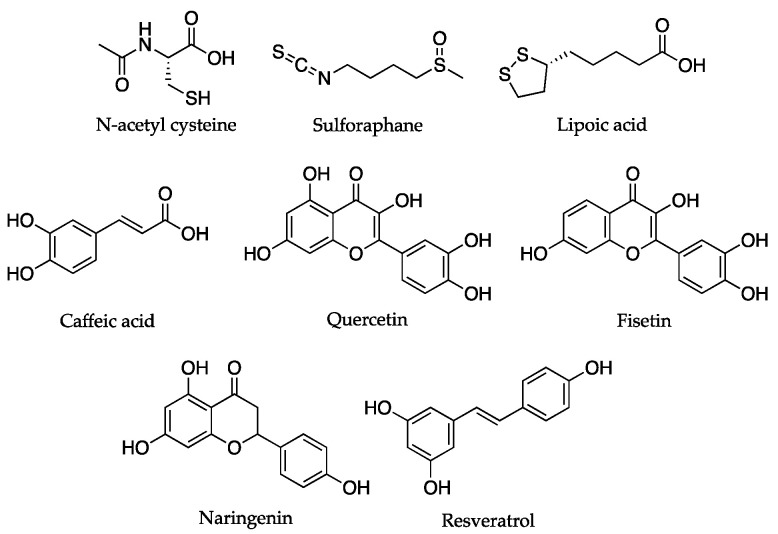
Chemical structures of natural compounds that directly or indirectly act as antioxidants, and which target mitochondrial decay.

Visioli et al. focused their attention on (poly)phenolic compounds of natural origin; these comprise a class of molecules that are considered to be “dietary antioxidants”. This is because they can act by terminating ROS and reactive nitrogen species, thus preventing damage. Importantly, some reports demonstrated that these compounds accumulate in mitochondria in vitro. The molecules in this class (e.g., resveratrol), ameliorate mitochondrial functions via a combination of mechanisms such as the stimulation of sirtuin and AMPK, the activation of Nrf2, direct antioxidant activity, and the modulation of mitochondrial-mediated programmed cell death [58]. The chemical structures of the compounds cited in this paragraph are reported in Figure 5.

### 4.4. Dietary Compounds Acting through Mixed Mechanisms

In a recent study, Bjørklund et al. reviewed the anti-aging properties of natural compounds from different chemical classes which exhibit their effects via a combination of several mechanisms [3]. Dietary phytochemicals are secondary plant metabolites that are present in foods such as vegetables and fruits, and they are also present in beverages [61]. It has been calculated that more than 1 g of phytochemicals is ingested in a normal diet; polyphenols comprise the largest portion of ingested phytochemicals [62]. Moreover, such compounds can be classified into the following major categories: carbohydrates, lipids, phenolic compounds, terpenoids, alkaloids, and other nitrogen-containing compounds [61,63]. Furthermore, Si and Liu reviewed the mechanisms through which this chemically diverse group of compounds can exert their anti-aging activities [61]. Luo et al. recently presented an overview of lifespan-extending natural molecules [64]. In this section of the review, we focus on the potential rejuvenating effects of dietary compounds.

Polyphenols are a wide class of molecules comprising phenolic acids, flavonoids, stilbenes, lignans, and other phenolic compounds [65]. Resveratrol (Figure 5) is a naturally occurring derivative, which has been mentioned previously, that can target cell proliferation and aging through different mechanisms [66,67,68,69,70,71]. In particular, this compound has been shown to extend lifespans in invertebrates, and it has improved biomarkers relating to longevity in mice [58]. Concerning the reported molecular mechanisms, through which, resveratrol is potentially able to induce rejuvenation, activating SIRT1 has been proposed. This would lead to improved mitochondrial function and increased lifespan in animals [3]. More specifically, concerning its effect on aging clocks, it has been noted that resveratrol can significantly decrease age-related parameters in animal models, including genetic instability. In another study, resveratrol prevented the appearance of morphological changes in the cell that are correlated with age, thus protecting DNA from oxidative damage and reducing the generation of acetylated forms of histones [72]. Nevertheless, it must be noted that the role of resveratrol is a rather debated issue. More specifically, another report showed that this compound, although it can preserve vascular function in animal models, does not extend lifespan in rats [73]. This result was also confirmed by Pearson et al., who observed that resveratrol did not extend lifespans in mice [74]. It must be also stressed that resveratrol has been reported to act on age-related conditions that target several pathways, including those that are involved in inflammation processes [75]. Lifespan extension may therefore be connected with both anti-aging and rejuvenating properties. Additionally, it has been shown that resveratrol shows contradictory effects depending on the doses used, a feature which is not desirable for a drug-like compound; thus, its interaction with other drugs, its action as a thyroid disruptor [76], and the manner in which it reduces glutathione levels, are all features which contrast with its use as an antiaging agent.

**Figure 6:**
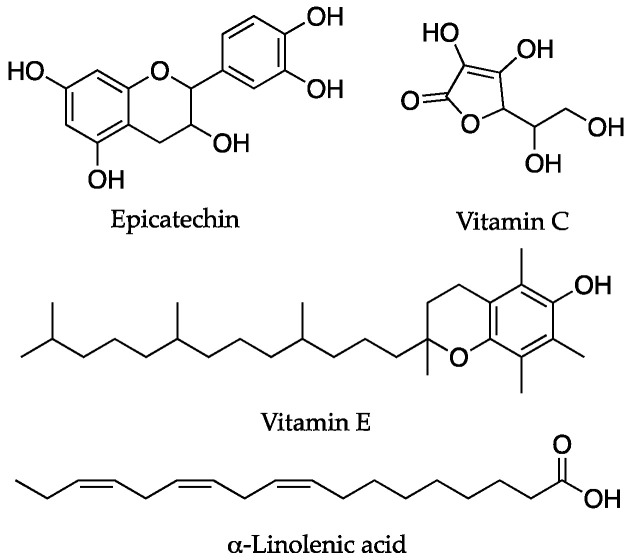
Chemical structures of dietary compounds that act as antioxidants through mixed mechanisms to exert their anti-aging or rejuvenation effects.

On the other hand, epicatechin (Figure 6), a compound present in several foods, including tea, cocoa, and its derivatives, is another polyphenol that has been studied for its rejuvenating effects [64]. Concerning its targeted mechanism, epicatechin acts on inflammation pathways, glutathione levels, superoxide dismutase activity, and it affects the IGF-1 pathway. As a result, epicatechin treatment led to lifespan extension in *Drosophila* and mouse models [77]. Moreover, McDonald et al. recently reported the positive effects of epicatechin on tissue biomarkers, which is indicative of mitochondrial biogenesis and muscle regeneration in vivo, thus further supporting its potential role in rejuvenation processes [78].

Vitamins also represent a source of lifespan-improving dietary compounds. In particular, vitamin C and vitamin E (Figure 6) show antioxidant activity, and they may act synergically with other antioxidants [3]. Most importantly, in addition to the well-known direct antioxidant effects, the role of vitamin E on gene expression has also been reported. This aspect is the most relevant as it represents an alternative, antioxidant activity-independent anti-aging mechanism [79,80].

Polyunsaturated fatty acids (PUFAs) comprise another category of relevant compounds involved in the regulation of aging. In particular, as well as their anti-inflammatory effects, omega-3 PUFAs, including α-linolenic acid (Figure 6), increase the levels of the signaling factors that contribute to plasticity, thus increasing hippocampal neurogenesis and enhancing dendritic synaptic spines; this leads to improved cognitive activity and the prevention of degeneration [81,82].

The anti-aging and rejuvenating effects of the compounds mentioned in this section are shown in Table 1.

It must be noted that natural compounds, and polyphenols in particular, as highlighted by Bjørklund et al., often appear to exert their activity as anti-aging molecules rather than through a true rejuvenation approach; this is despite the fact that these compounds are known for their antioxidant activity and their limitations in terms of bioavailability [3]. This is also evident in Table 1, which highlights how only some of the compounds that affect lifespan act through true rejuvenation mechanisms. Nevertheless, there is increasing evidence in support of the role that dietary compounds play with regard to molecular mechanisms and the manner in which they mediate rejuvenation. These mechanisms influence lifespan-modulating miRNA and mitochondrial biogenesis, processes which have recently been explored in the literature [64,78].

## 5. Conclusions and Future Directions

As is evident in the reports examined in this review, the possibility of achieving rejuvenation through interventions based on the use of small molecules relies on several complex factors. This is due to the fact that a combination of strategies targeting several pathways at the same time is needed to achieve the reversal of aging. It must be also noted that antioxidant molecules, and dietary compounds in particular, are among the most studied molecules in this field.

As a first step in the development of a rejuvenation approach based on small molecules, it must be clarified whether some cellular players (e.g., G4s and mitochondria) are “friends or foes”. This is because contradictory reports on their role, and that of the compounds targeting them, are present in the literature. Once this is implemented, more insights into the mechanisms driving anti-aging and rejuvenation processes, and possibly in vivo evidence, will be provided; however, there will still be a need to develop effective and selective interventions through compound optimization, bioavailability improvements, and delivery strategies.

Moreover, although most current contributions report on single-tissue improvements, further research is needed to develop a rejuvenation process that extends to a whole organism, and that can be measured through increasingly accurate aging clocks. In fact, at this stage, it may be premature to draw results from this experimental evidence, as there is also the need for a clear dissection between advanced age and “setting the clocks backwards”; thus true rejuvenation can only be achieved by fully understanding the molecular mechanisms that drive aging processes, that which arrests those processes, and even, that which reverses the aging process [83].

With this in mind, the findings reviewed in this study confirm the constantly increasing interest in this topic, and they pave the way for the advancement of knowledge in the field of rejuvenation.

## Figures and Tables

**Table 1:** Overview of the main anti-aging and rejuvenating effects of the compounds cited in this section, and their molecular mechanisms.

Compounds	Targeted Mechanisms	Anti-Aging Effects	Rejuvenating Effects	Experimental Model	References
Growth hormone	GH/IGF-1 pathway,		Decrease in epigenetic age	Mouse, human	[35,36]
Epicatechin	increased glutathione, superoxide dismutase, IGF-1 pathway, protein kinase-α activity, mithocondrial biogenesis		Improvement in tissue biomarkers, increased lifespan	*Drosophila*, mouse, human	[77,78]
Hesperetin	CISD2 activation	Decrease in muscular aging	Interference with gene expression	Mouse	[45,46]
Other polyphenols	Antioxidant activity, miRNA targeting	Reduction in the accumulation of senescent cells and in the expression of oxidative stress markers		*Caenorhabditis elegans*, *Drosophila*, rodents	[3,64]
PUFAs	Increase in the levels of signaling factors that are involved in neuronal plasticity	Improved cognitive activity and prevention of degeneration	Hippocampal neurogenesis	Mouse	[81,82]
Resveratrol	SIRT1	Prevention of DNA damage	Improvement in longevity of biomarkers	MRC5 human fibroblasts, invertebrates, mouse, rat	[3,58]
Sulforaphane, lipoic acid, caffeic acid, quercetin, fisetin, and naringenin	Nrf2, ROS	Mitochondrial biogenesis, mitophagy, restoration of metabolites		HepG2 cells, HHL-5 cells, neurons, mouse, rat	[58]
Vitamins (C, E)	Direct gene expression, antioxidant activity	Anti-aging activity, prevention of ROS damage		Cultured hepatocytes	[79,80]
